# Sepsis: Personalized Medicine Utilizing ‘Omic’ Technologies—A Paradigm Shift?

**DOI:** 10.3390/healthcare6030111

**Published:** 2018-09-07

**Authors:** Theis Skovsgaard Itenov, Daniel D. Murray, Jens Ulrik Stæhr Jensen

**Affiliations:** 1 PERSIMUNE, Rigshospitalet, Copenhagen DK-2100, Denmark; daniel.dawson.murray@regionh.dk (D.D.M.); jens.ulrik.jensen@regionh.dk (J.U.S.J.); 2 Department of Internal Medicine C, Respiratory Medicine Section, Herlev-Gentofte Hospital, Hellerup DK-2900, Denmark

**Keywords:** sepsis, systems biology, transitional research, metabolomics, genomics, transcriptomics

## Abstract

Sepsis has over the years proven a considerable challenge to physicians and researchers. Numerous pharmacological and non-pharmacological interventions have been tested in trials, but have unfortunately failed to improve the general prognosis. This has led to the speculation that the sepsis population may be too heterogeneous to be targeted with the traditional one treatment suits all’ approach. Recent advances in genetic and biochemical analyses now allow genotyping and biochemical characterisation of large groups of patients via the ‘omics’ technologies. These new opportunities could lead to a paradigm shift in the approach to sepsis towards personalised treatments with interventions targeted towards specific pathophysiological mechanisms activated in the patient. In this article, we review the potentials and pitfalls of using new advanced technologies to deepen our understanding of the clinical syndrome of sepsis.

## 1. Introduction: Sepsis—One Disease?

Sepsis is defined as a dysregulated host response to infection that may cause organ damage [1]. Sepsis is a leading cause of infection-related death worldwide and sepsis survivors often suffer from complications, reducing self-rated health and daily function [2]. Despite substantial research focus the pathophysiology remains unclear which is reflected in the clinical definition of the syndrome [1]. While this clinical definition has made it possible to compare incidence and outcomes across centres and regions, it also has the back side that several profoundly distinct pathophysiologies could lead to this rather non-specific clinical presentation. Patients suffering very different diseases are thus currently classified together, i.e., circulatory failure following major bowel surgery and patients with pneumonia, conditions that need differentiated therapy in almost all aspects. Nevertheless, this may only be the tip of the iceberg. The pathophysiologies leading to the systemic coagulation, endothelial leakage and circulatory collapse observed in septic shock may be different at a systemic and cellular level [3], and this is, almost certainly, influenced by, among other factors, host genomics, natural microbiome of the host and immune constitution.

During the last decade, several interventions have been investigated in septic patients, including administration of activated protein C, tight glycaemic control with insulin, early targeted fluid resuscitation, and mild induced hypothermia [4,5,6,7,8]. Few have proved beneficial in primary studies, and none in confirmatory trials. Although each of these interventions may have failed for several reasons, it may also be a consequence of the fact that we are unaware of the actual pathophysiology of the individual patient. Further, it is striking that all the above interventions were supported by a large base of data from cell line, animal or human experimental sepsis models. Thus, in vitro models have in general failed and animal studies have proven, for many reasons, to give very unprecise estimates of human sepsis pathophysiology.

Recent clinical studies have also demonstrated that biomarkers traditionally used to classify a patient’s degree of organ failure may only capture some aspects of organ failure, leaving groups of patients falsely classified as not suffering an organ failure or vice versa [9,10,11].

In summary, these findings, and especially decades of failed empiric trials, make it less likely that any intervention will work on the entire sepsis population as defined by the current definition, and the classic approach of cell studies and animal studies as rationale for new interventions in this field should be abandoned. We need to build our understanding of pathophysiology and potential for treatment from studies of humans. These new insights make it necessary to develop methods to study the individual processes taking place in the individual patient, and to design trials based on this. As reviewed recently, interpretation of these novel bio-analyses is key and complicated [12]. Eventually these methods will form a solid basis for selection of patients for certain diagnostic tests and for highly individualized interventions. Omics technologies could prove such a tool, but many challenges need to be overcome before this strategy becomes everyday clinical practice. This review is about these challenges and the perspectives that “omics” analyses can provide modern sepsis science and treatment practice.

## 2. The ’Omics’—What Can We Learn and What Are the Challenges

The relatively new field of omics profiling refers to the systematic measurement of an entire class of biochemical species, i.e., DNA, RNA lipids or small metabolites. Omics can be measured at a single time-point to give a snap-shot of an individual’s current biochemical state or longitudinally to determine how particular molecular pathways change over-time. With the advent and expansion of genomics technologies in the early to late 2000s it was hoped that our understanding of the causes of many common diseases would be explained by alterations in host-genetics. However, by the end of this decade it became clear that while important, host genetics could only reveal part of the disease risk and that environmental and other factors (i.e., microbiome) also play a role in driving pathogenic changes [13,14,15,16]. Therefore, to get a clearer picture of many diseases, it was essential to widen the lens and analyse the entire system through a variety of different omics techniques, with the main aim being to identify either inherited risk factors or alterations in biochemical processes that lead to disease. The major leap from traditional biochemical analysis to ‘omics’ technologies is the ability to quantify the entire state of a human with regards to genes, RNA, proteins, metabolites etc., whereas previous approaches have been more or less limited to single or few molecules. Omics technologies, on the other hand, allow for the measurement of anywhere from a few hundred analytes (some metabolomics and proteomics approaches) to millions of analytes (genomics approaches). This makes it possible to critically appraise entire pathways of disease pathogenesis rather than single molecule associations. Identification of these pathways can then be used to provide critical information about disease sub-types or the pathogenesis of specific clinical outcomes, allowing for the identification and stratification of individuals or groups based on the risk of a particular outcome or a predicted response to therapy. These omics profilings assess a variety of diverse biochemical systems and include (but are not limited to):

*(a) Genomics*: assessment of inherited genetic material or DNA mutations in somatic cells. Variations in DNA are measured by investigating the presence or absence of common single nucleotide polymorphisms (SNPs) (measured via SNP arrays [17]), the entirety of an individual’s exomes (whole exome sequencing [18]) or genome (whole genome sequencing [19]). Any cellular material containing DNA can be used to assess the inherited genome. Specific cellular subsets can also be assessed for somatic mutations.

*(b) Epigenomics*: A systematic measurement of all epigenetic changes to a person’s genome or histone proteins. Epigenetic changes (including DNA methylation, chromatin accessibility and histone modifications) control which genes are expressed in a particular cell type. Epigenetic modifications vary between cell-types and must be measured using a variety of methods in order to capture the different epigenetic changes (summarised in [20]).

*(c) Transcriptomics* (also known as gene expression analysis): an assessment of an individual’s transcribed RNA in blood or cells at a given time. Measured to give an indication of which genes are being expressed in a specific cell type or system. Assessed using micro-arrays or RNA-Seq technology [21].

*(d) Proteomics*: assessment of the composition of proteins in blood or cells at a given time. Measured to give an indication of the translated proteins present in a particular cell-type, system or biological fluid. Can also indicate post-translational modifications (i.e., phosphorylation, glycosylation, acetylation and oxidation). Predominantly measured using a variety of mass spectrometry methods [22,23].

*(e) Metabolomics*: assessment of all the metabolites in a particular system. Metabolites are all the small biochemical components and by-products of the physiological processes in the human body, and can be of human or microbial origin. Measured to give an indication of the biochemical pathways that are occurring in a specific cell-type, system or biological fluid. Predominantly measured using a variety of mass spectrometry [24] or nuclear magnetic resonance spectroscopy methods [25].

## 3. How to Apply Omics in Sepsis Research

The knowledge gained by successful omics profiling is already being implemented into clinical practice, particularly in oncology—with genetic and other molecular sub-typing used to identify specific disease sub-groups and to tailor treatment strategies to the individual [26,27,28,29]. In sepsis research, omics technologies are also showing promise [30]. Studies have identified potential subgroups of patients with sepsis based on transcriptomics [31,32,33] and metabolomics [34], as well as genetic risk factors for specific sepsis outcomes [35,36]. Further, genetic risk scores have been developed and validated in external populations [37]. However, the studies are difficult to compare due variations in population and analysis technique, reflecting an evolving new science field—bioinformatics. In contrast to the classic statistical field comparing exposure to outcome, bioinformatics focusses on understanding complex and dynamic systems. Bioinformatics is an essential tool if the large potential of omics data is to be realized, but also requires that clinicians and researchers in general adapt to new metrics and nomenclature. Further, the evolving nature of the field also leaves a risk of difficulty in reproducing the results and formal interpretation of results. Put in plain words, it may currently be difficult to distinguish if a finding is biology, math or neither. Thus, although the area has an enormous potential to improve management of patients, so far, no comprehensive model has been developed for sepsis patients to consistently characterize the pathways activated in specific patients. Consequently, focus is currently aimed on improving reproducibility in bioanalysis and in the development of models that are (more) easily interpreted. These are considerable challenges and large efforts are currently being put into finding plausible solutions.

The advancement, expansion and subsequent reduction in price of omics profiling technology have resulted in a significantly increased access of these profiling techniques for groups that have access to clinical samples, but not necessarily the in-house expertise required to analyse or interpret them. As detailed in the previous section, this is a critical point to overcome as omics analyses are becoming increasingly data-rich and require effective collaboration and communication between bioinformaticians, biologists, statisticians and clinicians to analyse and interpret omics results effectively, and then finally incorporate these results into the clinical setting where they can be fully assessed for clinical utility.

Another major issue, when it comes to designing effective omics studies in the context of sepsis, is the issue of sample size. The large number of markers measured using any one omics technology and small contributing effect size of many of the analytes, mean that without sufficient sample sizes for discovery and validation cohorts the risk of both type 1 and 2 errors are high. In consequence, resources may be wasted or potentially unethical and dangerous results may enter the clinical setting if spurious results are allowed to persist [38]. As no single site is likely to be able to recruit enough patients, nor have the relevant expertise, machinery or funding to perform the variety of omics analyses that may be needed to elucidate sepsis subtypes, it is clear that a collaborative approach to omics based sepsis research must be pursued. This sort of approach is already underway in asthma research (a disease that is also complicated by a heterogenous pathogenesis) and producing promising results [39]. Of particular interest is the U-BIOPRED (Unbiased BIOmarkers in PREDiction of respiratory disease outcomes) initiative. U-BIOPRED is a multicentre collaborative research program aimed at identifying asthma endotypes using novel omics techniques and traditional clinical measurements [40]. This initiative has already succeeded in identifying potential asthma endotypes based on transcriptomics data [41], identified genomic characteristics associated with various inflammatory phenotypes [42] as well as defined transcriptomic characteristics of clinically defined phenotypes [43]. This multi-institute approach should be considered as a model for sepsis research initiatives moving forward, as both a discovery phase for identifying clinically relevant phenotypes and/or biomarkers, but also as a platform for implementing any observations into a clinical setting. Such a collaborative research would also facilitate the standardisation and sharing of research methods, expertise (both analytical and clinical), as well as descriptions/definitions of clinical outcomes and causes of death, in a similar way to what has been done in HIV and transplant research [44,45]. The most common approach today is post hoc collaborations across centres with biomaterial and data from previous investigations. This has proven a viable solution, but care must be taken when using existing biobanks and databases. One should critically appraise how regional or site variations in inclusion and outcome ascertainment may influence results and make direct interpretation of data difficult. Unambiguous definitions of populations, phenotypes and outcomes may prove to be the key to producing stable findings.

## 4. Towards Personalized Treatment of Sepsis

It is clear from previous sections that omics technologies can and will probably play a significant role in sepsis research moving forward. However, what is not clear is how these technologies will necessarily lead to clinical benefit for the sepsis patient. This section will suggest some strategies to integrate both clinical and omics interrogation of sepsis patients with an eye on improving clinical outcome in these individuals.

The analysis of omics technologies is often viewed as a black-box, whereby samples are put in on one end and biologically relevant clusters or associations come out the other end. However, without clinical understanding of the underlying disease, a clear biological question and an adequately powered sample size there is a significant risk of producing results and literature that have little to no biological or clinical relevance. Several strategies could be envisioned:

Some phenotypes are well-recognized as being associated with a particular adverse outcome. Understanding pathways leading to an increased incidence of these could prove valuable in identifying targets for intervention. These include, but are not limited to, endothelial damage, sepsis associated liver damage, sepsis associated lung damage and acute kidney injury. As such, it is critical that clinical input occurs prior to initiation of omics studies and relevant phenotypes are defined from the get-go. Previous approaches often solely focus on mortality as the phenotype of interest (i.e., dead or alive at follow-up). While this approach would be valid for a homogeneous disease, as discussed previously it is more likely that sepsis consists of a variety of different pathogeneses. These different pathogeneses may then lead to mortality in a variety of ways. Grouping and analysing these together risks masking the signal of each individual aetiology and should be avoided where possible.

A second approach, as have been previously attempted, is to try to identify groups of patients that share a common pathophysiology. This is achieved through methods commonly referred to as unsupervised learning. In unsupervised learning, clusters of patients are derived based on similarities across many different biologic characteristics. In these types of studies, the question becomes which groups to include and analyse: (a) Patients meeting the current definition of sepsis to understand the diversity of pathophysiologies across several different infectious sources and different organ failures; or (b) patients with a specific infection to understand the pathophysiologies that cause the variation in outcome and clinical presentation? Both approaches should be considered as presenting different aspect of the disease.

Just as clinical input should be sought before designing the omics aspect of the study, so should statistical, biological and bioinformatic expertise sought prior to initiation of any study. This is to ensure that the omics platforms and analysis techniques chosen can adequately answer the biological question posed. There are also critical (particularly with analyses of the genome), regulatory and even storage steps that need to be carefully considered prior to the initiation of any clinical study involving the collection of clinical samples and omics analyses. Traditional analyses of exposure and outcome, may not fully utilise the power of the combined data—and may lead to overly conservative estimates—or give altogether wrong answers. As mentioned in previous sections there are a variety of different omics platforms available and these can be performed in isolation or in a complimentary fashion.

Due to the reduction in costs of technology and standardised analysis pipelines analyses main omics tool used in research is often genomics. Although this technology presents somewhat of a problem in sepsis research, as it is likely that the heritability of the immune response to sepsis is low. If so, no single genetic variant will be responsible for the clinical outcomes seen in sepsis patients, but rather by a multitude of weakly associated variants. These weakly associated variants are likely to vary between individuals, but still lead to the same phenotype by altering expression of a particular gene or pathway. Traditional genome wide association studies are unlikely to detect these variants, however, other types of analyses (i.e., gene-set analyses (GSA)) have been developed to overcome these issues [46]. In GSA, SNPs are first annotated to genes and genes similarly annotated to pathways (also known as gene-sets). Once SNPs have been aggregated and annotated to gene-sets the association with, or enrichment of, SNPs within a particular gene-set is tested against a phenotype—asking the question whether sets of SNPs (when aggregated in genes or gene-sets) are more associated with a particular phenotype than would be expected by chance. This approach has been shown to be a reliable methodology for analysing diseases with complex pathogenesis and can also be applied to rare variants [47]. Additionally, as GSA bypasses the relatively small effect size of individual SNPs by aggregating them to genes and gene sets, this allows for increased power for identifying biological associations in smaller sample sizes [48].

While there may be some heritable aspect of the host response in sepsis, it is also likely that environmental factors will also influence how an individual or groups of individuals respond to infection. As such it is unlikely that analysing the genome in isolation will provide a true understanding of sepsis pathogenesis and a multi-omics strategy should be pursued wherever possible. Incorporating additional omics analyses of down-stream biochemical groups (i.e., transcriptomics, proteomics or metabolomics) enables a real-time snapshot of the cellular and extra-cellular signalling pathways that are up and down-regulated in different clinical sub-groups, while also providing additional weight to observed genetic variants (by measuring down-stream gene products). Ideally, these pathway style analyses can also reveal critical biomarkers that will be able to stratify the clinical sub-groups, making real-time stratification in the clinical setting simpler.

**Figure 1:**
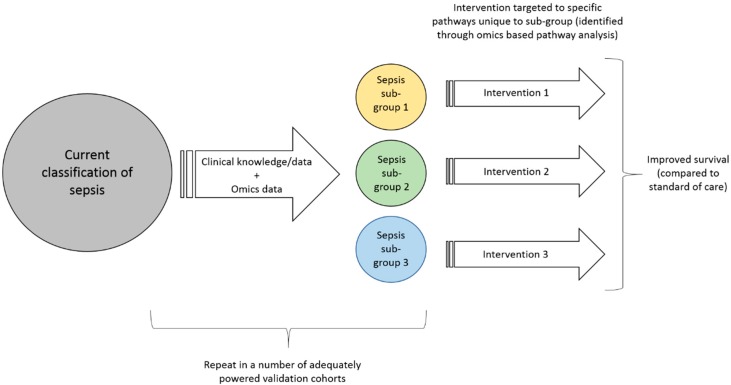
A suggestion for how to translate discoveries from ‘omics’ data to clinical practice.

Once these clinical sub-groups are more clearly defined through enrichment with omics based biomarkers (including confirmation in adequately powered validation studies), these biomarkers must undergo the rigorous quality control required to enable omics based guidance of patient care in clinical trials [49]. Further, the technologies must be transformed into tools that can be used in a clinical setting. For a disease such as sepsis that affect many patients, evolve quickly and likely need interventions to be started shortly after presentation, this includes high internal validity, short turn-around time from sampling to result and substantial cost-reduction. Only then can the focus can shift towards designing interventional strategies that are tailored to the specific sub-groups. These interventional strategies would ideally be tailored to the specific aetiology of the sub-group (as discovered using omics based pathway analysis) and may require new drug discovery (of which omics can also play a role [50,51,52]) or other interventional strategies. With this more targeted treatment approach we would then expect to see a clear reduction in overall sepsis mortality, as well as the more specific sub-group outcomes, Figure 1.

## 5. Conclusions

In conclusion, sepsis research is at a critical point in time. The discovery path from previous trials and animal models has proven futile and should be abandoned. New approaches must be considered and have been made technically possible. We propose to utilize new systems biology approaches to understand if sepsis patients are grouped beyond the simple clinical definition used today. New high throughput omic technologies will be crucial in understanding the pathophysiological subgroups of patients that have potential to respond to a given treatment, thus to improve prognosis and increase the understanding of this complex disease entity.

## References

[1] Singer M., Deutschman C.S., Seymour C.W., Shankar-Hari M., Annane D., Bauer M., Bellomo R., Bernard G.R., Chiche J.-D., Coopersmith C.M. (2016). The third international consensus definitions for sepsis and septic shock (Sepsis-3). JAMA.

[2] Vejen M., Bjorner J.B., Bestle M.H., Lindhardt A., Jensen J.U. (2017). Self-rated health as a predictor of death after two years: The importance of physical and mental wellbeing postintensive care. BioMed Res. Int..

[3] Angus D.C., Van der Poll T. (2013). Severe sepsis and septic shock. N. Engl. J. Med..

[4] Ranieri V.M., Thompson T., Barie P.S., Dhainaut J.-F., Douglas I.S., Finfer S., Gårdlund B., Marshall J.C., Rhodes A., Artigas A. (2012). Drotrecogin alfa (activated) in adults with septic shock. N. Engl. J. Med..

[5] Van den Berghe G., Wouters P., Weekers F., Verwaest C., Bruyninckx F., Schetz M., Vlasselaers D., Ferdinande P., Lauwers P., Bouillon R. (2001). Intensive insulin therapy in critically ill patients. N. Engl. J. Med..

[6] The NICE-SUGAR Study Investigators (2009). Intensive versus conventional glucose control in critically ill patients. N. Engl. J. Med..

[7] The ARISE Investigators, The ANZICS Clinical Trials Group (2014). Goal-directed resuscitation for patients with early septic shock. N. Engl. J. Med..

[8] Itenov T.S., Johansen M.E., Bestle M., Thormar K., Hein L., Gyldensted L., Lindhardt A., Christensen H., Estrup S., Pedersen H.P. (2018). Induced hypothermia in patients with septic shock and respiratory failure (CASS): A randomised, controlled, open-label trial. Lancet Respir. Med..

[9] The “Procalcitonin and Survival Study” Study Group (2017). Endothelial damage signals refractory acute kidney injury in critically ill patients. SHOCK.

[10] Johansen M.E., Johansson P.I., Ostrowski S.R., Bestle M.H., Hein L., Jensen A.L.G., Søe-Jensen P., Andersen M.H., Steensen M., Mohr T. (2015). Profound endothelial damage predicts impending organ failure and death in sepsis. Semin. Thromb. Hemost..

[11] Jensen J.-U.S., Itenov T.S., Thormar K.M., Hein L., Mohr T.T., Andersen M.H., Løken J., Tousi H., Lundgren B., Boesen H.C. (2016). Prediction of non-recovery from ventilator-demanding acute respiratory failure, ARDS and death using lung damage biomarkers: Data from a 1200-patient critical care randomized trial. Ann. Intensive Care.

[12] Sweeney T.E., Wong H.R. (2016). Risk stratification and prognosis in sepsis: What have we learned from microarrays. Clin. Chest Med..

[13] Maher B. (2008). Personal genomes: The case of the missing heritability. Nature.

[14] Hall S.S. (2010). Revolution postponed. Sci. Am..

[15] Cho I., Blaser M.J. (2012). The human microbiome: At the interface of health and disease. Nat. Rev. Genet..

[16] Feil R., Fraga M.F. (2012). Epigenetics and the environment: Emerging patterns and implications. Nat. Rev. Genet..

[17] LaFramboise T. (2009). Single nucleotide polymorphism arrays: A decade of biological, computational and technological advances. Nucleic Acids Res..

[18] Bamshad M.J., Ng S.B., Bigham A.W., Tabor H.K., Emond M.J., Nickerson D.A., Shendure J. (2011). Exome sequencing as a tool for Mendelian disease gene discovery. Nat. Rev. Genet..

[19] Cirulli E.T., Goldstein D.B. (2010). Uncovering the roles of rare variants in common disease through whole-genome sequencing. Nat. Rev. Genet..

[20] Roadmap E.C., Kundaje A., Meuleman W., Ernst J., Bilenky M., Yen A., Heravi-Moussavi A., Kheradpour P., Zhang Z., Wang J. (2015). Integrative analysis of 111 reference human epigenomes. Nature.

[21] Wang Z., Gerstein M., Snyder M. (2009). RNA-Seq: A revolutionary tool for transcriptomics. Nat. Rev. Genet..

[22] Geyer P.E., Holdt L.M., Teupser D., Mann M. (2017). Revisiting biomarker discovery by plasma proteomics. Mol. Syst. Biol..

[23] Larance M., Lamond A.I. (2015). Multidimensional proteomics for cell biology. Nat. Rev. Mol. Cell Biol..

[24] Gika H.G., Theodoridis G.A., Plumb R.S., Wilson I.D. (2014). Current practice of liquid chromatography-mass spectrometry in metabolomics and metabonomics. J. Pharm. Biomed. Anal..

[25] Markley J.L., Brüschweiler R., Edison A.S., Eghbalnia H.R., Powers R., Raftery D., Wishart D.S. (2017). The future of NMR-based metabolomics. Curr. Opin. Biotechnol..

[26] Dienstmann R., Vermeulen L., Guinney J., Kopetz S., Tejpar S., Tabernero J. (2017). Consensus molecular subtypes and the evolution of precision medicine in colorectal cancer. Nat. Rev. Cancer.

[27] Mody R.J., Prensner J.R., Everett J., Parsons D.W., Chinnaiyan A.M. (2017). Precision medicine in pediatric oncology: Lessons learned and next steps. Pediatr. Blood Cancer.

[28] Masters G.A., Johnson D.H., Temin S. (2016). Systemic therapy for stage IV non-small-cell lung cancer: American society of clinical oncology clinical practice guideline update. J. Oncol. Pract..

[29] Coates A.S., Winer E.P., Goldhirsch A., Gelber R.D., Gnant M., Piccart-Gebhart M., Thürlimann B., Senn H.-J., Members P., André F. (2015). Tailoring therapies—Improving the management of early breast cancer: St Gallen International Expert Consensus on the Primary Therapy of Early Breast Cancer 2015. Ann. Oncol..

[30] Lydon E.C., Ko E.R., Tsalik E.L. (2018). The host response as a tool for infectious disease diagnosis and management. Expert Rev. Mol. Diagn..

[31] Scicluna B.P., Vught L.A., Zwinderman A.H., Wiewel M.A., Davenport E.E., Burnham K.L., Nürnberg P., Schultz M.J., Horn J., Cremer O.L. (2017). Classification of patients with sepsis according to blood genomic endotype: A prospective cohort study. Lancet Respir. Med..

[32] Sweeney T.E., Azad T.D., Donato M., Haynes W.A., Perumal T.M., Henao R., Bermejo-Martin J.F., Almansa R., Tamayo E., Howrylak J.A. (2018). Unsupervised analysis of transcriptomics in bacterial sepsis across multiple datasets reveals three robust clusters. Crit. Care Med..

[33] Wong H.R., Cvijanovich N., Lin R., Allen G.L., Thomas N.J., Willson D.F., Freishtat R.J., Anas N., Meyer K., Checchia P.A. (2009). Identification of pediatric septic shock subclasses based on genome-wide expression profiling. BMC Med..

[34] Langley R.J., Tsalik E.L., Van Velkinburgh J.C., Glickman S.W., Rice B.J., Wang C., Chen B., Carin L., Suarez A., Mohney R.P. (2013). An integrated clinico-metabolomic model improves prediction of death in sepsis. Sci. Transl. Med..

[35] Rautanen A., Mills T.C., Gordon A.C., Hutton P., Steffens M., Nuamah R., Chiche P.-D., Parks T., Chapman S.J., Davenport E.E. (2015). Genome-wide association study of survival from sepsis due to pneumonia: An observational cohort study. Lancet Respir. Med..

[36] Scherag A., Schöneweck F., Kesselmeier M., Taudien S., Platzer M., Felder M., Sponholz C., Rautanen A., Hill A.V.S., Hinds C.J. (2016). Genetic factors of the disease course after sepsis: A genome-wide study for 28 day mortality. EBioMedicine.

[37] Sweeney T.E., Perumal T.M., Henao R., Nichols M., Howrylak J.A., Choi A.M., Bermejo-Martin J.F., Almansa R., Tamayo E., Davenport E.E. (2018). A community approach to mortality prediction in sepsis via gene expression analysis. Nat. Commun..

[38] Krzywinski M., Altman N. (2013). Power and sample size. Nat. Methods.

[39] Svenningsen S., Nair P. (2017). Asthma endotypes and an overview of targeted therapy for asthma. Front. Med..

[40] Shaw D.E., Sousa A.R., Fowler S.J., Fleming L.J., Roberts G., Corfield J., Pandis I., Bansal A.T., Bel E.H., Auffray C. (2015). Clinical and inflammatory characteristics of the European U-BIOPRED adult severe asthma cohort. Eur. Respir. J..

[41] Bigler J., Boedigheimer M., Schofield J.P.R., Skipp P.J., Corfield J., Rowe A., Sousa A.R., Timour M., Twehues L., Hu X. (2017). A severe asthma disease signature from gene expression profiling of peripheral blood from U-BIOPRED cohorts. Am. J. Respir. Crit. Care Med..

[42] Wilson S.J., Ward J.A., Sousa A.R., Corfield J., Bansal A.T., De Meulder B., Lefaudeux D., Auffray C., Loza M.J., Baribaud F. (2016). Severe asthma exists despite suppressed tissue inflammation: Findings of the U-BIOPRED study. Eur. Respir. J..

[43] Loza M.J., Djukanovic R., Chung K.F., Horowitz D., Ma K., Branigan P., Barnathan E.S., Susulic V.S., Silkoff P.E., Sterk P.J. (2016). Validated and longitudinally stable asthma phenotypes based on cluster analysis of the ADEPT study. Respir. Res..

[44] Lifson A.R., Belloso W.H., Carey C., Davey R.T., Duprez D., El-Sadr W.M., Gatell J.M., Gey D.C., Hoy J.F., Krum E.A. (2008). Determination of the underlying cause of death in three multicenter international HIV clinical trials. HIV Clin. Trials.

[45] Wareham N.E., Cunha-Bang C.D., Borges A.H., Ekenberg C., Gerstoft J., Gustafsson F., Hansen D., Helleberg M., Heilmann C., Hillingsø J. (2018). Classification of death causes after transplantation (CLASS): Evaluation of methodology and initial results. Medicine.

[46] Visscher P.M., Brown M.A., McCarthy M.I., Yang J. (2012). Five years of GWAS discovery. Am. J. Hum. Genet..

[47] Kao P.Y.P., Leung K.H., Chan L.W.C., Yip S.P., Yap M.K.H. (2017). Pathway analysis of complex diseases for GWAS, extending to consider rare variants, multi-omics and interactions. Biochim. Biophys. Acta.

[48] Wang K., Li M., Hakonarson H. (2010). Analysing biological pathways in genome-wide association studies. Nat. Rev. Genet..

[49] McShane L.M., Cavenagh M.M., Lively T.G., Eberhard D.A., Bigbee W.L., Williams P.M., Mesirov J.P., Polley M.-Y.C., Kim K.Y., Tricoli J.V. (2013). Criteria for the use of omics-based predictors in clinical trials. Nature.

[50] Butcher E.C., Berg E.L., Kunkel E.J. (2004). Systems biology in drug discovery. Nat. Biotechnol..

[51] Wishart D.S. (2016). Emerging applications of metabolomics in drug discovery and precision medicine. Nat. Rev. Drug Discov..

[52] Dopazo J. (2014). Genomics and transcriptomics in drug discovery. Drug Discov. Today.

